# Multitasking dynamic contrast enhanced magnetic resonance imaging can accurately differentiate chronic pancreatitis from pancreatic ductal adenocarcinoma

**DOI:** 10.3389/fonc.2022.1007134

**Published:** 2023-01-06

**Authors:** Nan Wang, Srinivas Gaddam, Yibin Xie, Anthony G. Christodoulou, Chaowei Wu, Sen Ma, Zhaoyang Fan, Lixia Wang, Simon Lo, Andrew E. Hendifar, Stephen J. Pandol, Debiao Li

**Affiliations:** ^1^ Biomedical Imaging Research Institute, Cedars-Sinai Medical Center, Los Angeles, CA, United States; ^2^ The Karsh Division of Gastroenterology and Hepatology, Cedars Sinai Medical Center, Los Angeles, CA, United States; ^3^ Bioengineering Department, University of California, Los Angeles, Los Angeles, CA, United States; ^4^ Department of Radiology, Keck School of Medicine, University of Southern California, Los Angeles, Los Angeles, CA, United States; ^5^ Samuel Oschin Comprehensive Cancer Center, Cedars-Sinai Medical Center, Los Angeles, CA, United States

**Keywords:** quantitative imaging, dynamic contrast enhanced magnetic resonance imaging, microcirculation properties, Multitasking DCE, differential diagnosis of chronic pancreatitis and pancreatic ductal adenocarcinoma

## Abstract

**Background and aims:**

Accurate differentiation of chronic pancreatitis (CP) and pancreatic ductal adenocarcinoma (PDAC) is an area of unmet clinical need. In this study, a novel Multitasking dynamic contrast enhanced (DCE) magnetic resonance imaging (MRI) technique was used to quantitatively evaluate the microcirculation properties of pancreas in CP and PDAC and differentiate between them.

**Methods:**

The Multitasking DCE technique was able to acquire one 3D image per second during the passage of MRI contrast agent, allowing the quantitative estimation of microcirculation properties of tissue, including blood flow F_p_, plasma volume fraction v_p_, transfer constant K^trans^, and extravascular extracellular volume fraction v_e_. Receiver operating characteristic (ROC) analysis was performed to differentiate the CP pancreas, PDAC pancreas, normal control pancreas, PDAC tumor, PDAC upstream, and PDAC downstream. ROCs from quantitative analysis and conventional analysis were compared.

**Results:**

Fourteen PDAC patients, 8 CP patients and 20 healthy subjects were prospectively recruited. The combination of F_p_, v_p_, K^trans^, and v_e_ can differentiate CP versus PDAC pancreas with good AUC (AUC [95% CI] = 0.821 [0.654 – 0.988]), CP versus normal pancreas with excellent AUC (1.000 [1.000 – 1.000]), PDAC pancreas versus normal pancreas with excellent AUC (1.000 [1.000 – 1.000]), CP versus PDAC tumor with excellent AUC (1.000 [1.000 – 1.000]), CP versus PDAC downstream with excellent AUC (0.917 [0.795 – 1.000]), and CP versus PDAC upstream with fair AUC (0.722 [0.465 – 0.980]). This quantitative analysis outperformed conventional analysis in differentiation of each pair.

**Conclusion:**

Multitasking DCE MRI is a promising clinical tool that is capable of unbiased quantitative differentiation between CP from PDAC.

## Introduction

Pancreatic ductal adenocarcinoma (PDAC) is the third most common cause of cancer-related death in the United States with a poor 5-year survival rate of 9% (1). Currently the only curative treatment for PDAC is complete tumor resection, often in conjugation with adjuvant chemotherapy (2), where an accurate diagnosis at early stage is a prerequisite. Chronic pancreatitis is a fibrotic reaction of the pancreatic connective tissue due to an ongoing inflammation that can damage both endocrine and exocrine pancreas (3). The most worrisome complication of CP is the increased risk for developing PDAC, which can be 2.3 -18.5 folds higher (4–7). On the other hand, 10-20% of CP cases can be mass forming and mimic PDAC, which may cause misdiagnosis and overtreatment (8). PDAC is also likely to be associated with chronic obstructive pancreatitis in the upstream portion of the pancreas as a result of main pancreatic ductal obstruction by tumor (9).

Accurate differentiation of PDAC from CP is of great clinical importance for timely and precise treatment. However, this continues to be a challenging area due to the shared clinical signs, radiologic features, and morphologic appearance of the two diseases (10–13). Conventional imaging techniques including endoscopic ultrasound (EUS), PET/CT, and MRI cannot differentiate well between early PDAC and CP as the typical imaging features of CP (generalized parenchymal glandular atrophy, diffuse pancreatic calcifications, and dilation of the main pancreatic duct) can often be seen in PDAC (14, 15), resulting in reduced diagnostic accuracy. Even a fine needle biopsy (FNB) can be unreliable in this situation (16–18). This may result in further delay of diagnosis and treatment of PDAC or unnecessary surgery and exposure to complications of CP (11, 19).

In recent years, dynamic contrast enhanced (DCE) magnetic resonance imaging (MRI) has been an emerging tool for the clinical diagnosis of PDAC. Investigational studies also showed that DCE MRI may have a promising role in the diagnosis of CP and the differentiation of CP versus PDAC (20–23). It acquires a series of T1-weighted images during the injection and passage of gadolinium (Gd)-based contrast agent (CA). The changes of the signal intensity reflect the CA distribution within the tissue and the underlying microcirculation properties such as tissue blood flow, microvascular density, permeability, and extravascular extracellular space distribution. These microcirculation properties contain crucial information about disease characteristics, progression, and regression, and can be used for diagnosis and therapy monitoring (23–25).

However, DCE MRI has yet to fully realize its potential in the imaging of pancreas due to demanding technical challenges. Existing techniques cannot achieve adequate coverage and high spatiotemporal resolution at the same time. In clinical practice, T1-weighted images are usually acquired for four to six phases during the CA passage (referred as multi-phase MRI) (22, 26, 27), and each phase takes 15-20 seconds. In addition, the presence of respiratory motion and the need to hold breath makes the time intervals even larger, which is insufficient to quantify the microcirculation properties. Consequently, current diagnosis relies only on the morphological information of the pre- and post-contrast images, which are subject to coil positioning, and inter-scanner and inter-reader variability given its qualitative nature.

To overcome these limitations, our research group has developed a quantitative Multitasking DCE MRI technique (28) that has shown promise in the characterization of carotid atherosclerosis (29), PDAC (30), and breast cancer (31). Specifically for pancreas, this technique allows free-breathing acquisition, coverage of the entire abdomen, clinically sufficient spatial resolution, 1-second temporal resolution (one 3D image per second). With the high temporal resolution, Multitasking DCE is able to capture the contrast agent kinetics within the tissues, and thus to quantitatively evaluate the microcirculation properties. Our prior work has preliminarily demonstrated that Multitasking DCE MRI can produce high-quality image with free-breathing acquisition and characterize PDAC tissues (30). In this study, we aim to quantitatively evaluate the microcirculation properties of pancreas in CP and PDAC using Multitasking DCE, and to distinguish them with the quantitative parameters on an objective basis.

## Materials and methods

### Study population

The prospective study was approved by the local institutional review board and written informed consent was obtained from all participating subjects before the research imaging studies. The study was performed from February 2018 to June 2019 with PDAC patients, CP patients, and healthy volunteers. Among them, nineteen patients with PDAC, which was confirmed by histopathology obtained by EUS-guided FNB, were recruited to the study. All the PDAC patients received clinical CT within 1 week before the research MRI and were undergoing neoadjuvant chemotherapy at the time of the study. Patients were excluded for the following reasons: 1) prior surgical resections of PDAC; 2) intolerance to Gd-based contrast agent. Eight patients with definitive CP were recruited from an NIH-sponsored prospective cohort of patients with pancreatic disease (32). The inclusionary criteria were the clinical diagnosis of unequivocal CP (Cambridge grade >3). All these images were reviewed and confirmed to be CP by a radiologist as part of the PROCEED study (NCT03099850) (32). Healthy volunteers without a history of pancreas diseases or family history of pancreatic cancer were recruited as the normal control group. Subjects with noticeable pancreatic abnormality were excluded from the final analysis.

### MRI experiments

All subjects received the research MRI imaging on a 3-Tesla clinical MRI scanner (Biograph mMR, Siemens Medical Solutions, Erlangen, Germany) in head-first supine position with an 18-channel phase array surface coil. In the imaging session, a standard-of-care non-contrast protocol was first performed. It consisted of:

1) 3D T1-weighted gradient echo with Dixon fat suppression in axial orientation with parameters: 18-second breath-holding, flip angle = 9°, field of view (FOV) = 247 × 380 mm, acquisition matrix = 180 × 320, slice thickness = 3 mm, number of slices = 72.
2) Multi-slice T2-weighted single-shot turbo spin-echo in axial and coronal orientations with parameters: 42-second free-breathing, flip angle = 105°, FOV=226 × 330 mm, matrix =176 × 256, slice thickness = 5 mm, slice gap = 1 mm, number of slices = 46.
3) Multi-slice single-shot echo-planar diffusion-weighted imaging with parameters: 5-min free-breathing, *b*-values = 50, 400, and 800 s/mm^2^, FOV = 306 × 399 mm; matrix, 132 × 172, slice thickness = 6 mm, slice gap = 1 mm, number of slices = 50.
4) Multi-slice magnetic resonance cholangiopancreatography (MRCP): 10-min respiratory-triggering, flip angle = 100°, FOV = 300 × 300 mm, acquisition matrix = 384 × 384, slice thickness = 1 mm, number of slices = 80.
5) Multitasking DCE. It is a 10-min free-breathing acquisition of saturation-prepared gradient echo sequence with following parameters: saturation recovery time = 500 ms, flip angle = 10°, field of view (FOV) = 268 × 380 mm, acquisition matrix = 200 × 320, slice thickness = 3 mm, number of slices = 120. The Gd-based contrast agent (Gadavist, 0.1 mmol/kg, Bayer Schering Pharma) was administrated intravenously 3 minutes into the scan at a rate of 2 mL/s, followed by a 20 mL saline flush at the same rate. The reconstructed images have a temporal resolution of 1 second.

Detailed imaging parameters for the protocols are summarized in **Table 1**.

**Table 1:** List of imaging parameters.

parameters	T1W GRE	T2W HASTE	SS-EPI DWI	MRCP	Multitasking DCE
**Slice thickness (mm)**	3	5	6	1	3
**Slice resolution**	50%	N/A	N/A	N/A	50%
**Gap (mm)**	N/A	1	1	0	N/A
**Number of slices acquired**	72	86	50	80	120
**TR (ms)**	4.15	1000	4500	8903	5.60
**TE (ms)**	1.39/2.65(OP/IP)	99	47	701	2.45
**Number of averages**	1	1	6	1	1
**FOV (mm^2^)**	247×380	226×330	306×339	300×300	268×380
**Acquisition matrix**	180×320	176×256	132×172	384×384	200×320
**Flip angle (◦)**	9	105	90	100	10
**iPAT factor**	3	2	2	2	N/A
**Scan time**	18-second breath hold	42-second free-breathing	5-min free-breathing	10-min resp-triggered	10-min free-breathing

T1W GRE, T1-weighted gradient echo; T2W HASTE, T2-weighted single-shot turbo spin-echo; SS-EPI-DWI, single-shot echo-planar diffusion weighted imaging; MRCP, magnetic resonance cholangiopancreatography; N/A, Not applicable.

### Multitasking DCE reconstruction and quantitative DCE modeling

The reconstruction and quantitative analysis of Multitasking DCE images were processed off-line in MATLAB (R2018a, Mathworks, MA, USA). The details on the reconstruction have been described in Wang et al (30). In this work, the 3D Multitasking DCE images covering the entire abdomen were reconstructed at 6 respiratory states and the images of end-expiration were used for subsequent analysis. The reconstructed spatial resolution is 1.2 × 1.2 × 3.0 mm (3). The reconstructed temporal resolution is 1 second, leading to 600 dynamic T1 maps within the 10-minute acquisition.

With the dynamic T1 maps, the CA concentration can be directly calculated without approximation using the equation:


(1) $${\text{C}}_{\text{t}}(t_{d})=\frac{{\text{R}}_{1,\text{t}}(t_{d})-{\text{R}}_{1}(0)}{\gamma },$$

where *C*_t_ is the CA concentration in a certain tissue (any type of tissue within the FOV), *t_d_* is the DCE time points from 0 to 10 minutes at an interval of 1 second, *R*_1,t_ is the relaxation rate (1/*T*_1,t_) of the tissue, and *γ*= 4.0 L·mmol^-1^·s^-1^ is the relaxivity rate of Gadavist. The CA concentration in the arterial plasma *C*_p_, termed as arterial input function (AIF), can also be derived using Equation 1.

With the CA concentration of plasma *C*_p_ and of the target tissue *C*_t_, the two-compartment exchange model (33) was used to describe the contrast agent activities and estimate the microcirculation parameters including tissue plasma flow *F*_p_, fractional plasma volume *v*_p_, transfer constant *K* ^trans^, and extravascular extracellular faction *v*_e_. The microcirculation parameters were derived using following equations (31):


(2) $${\text{C}}_{\text{t}}({\text{t}}_{\text{d}})=\;{\text{F}}_{\text{p}}\cdot {\text{C}}_{\text{p}}({\text{t}}_{\text{d}})*\left({\text{Me}}^{-{\text{αt}}_{\text{d}}}+(1-\text{M}){\text{e}}^{-{\text{βt}}_{\text{d}}}\right),$$

(3) $$v_{\text{p}}=\frac{{\text{F}}_{\text{p}}}{\text{Mα}+(1-\text{M})\text{β}},\;\;{\text{K}}^{\text{trans}}={\text{F}}_{\text{p}}\frac{\text{M}(1-\text{M}){\left(\text{α}-\text{β}\right)}^{2}}{{\text{Mα}}^{2}+(1-\text{M}){\text{β}}^{2}},\;\;{\text{v}}_{\text{e}}=\;{\text{v}}_{\text{p}}\frac{\text{M}(1-\text{M}){\left(\text{α}-\text{β}\right)}^{2}}{\text{αβ}},$$

where * denotes convolution, and *M*, *α*, and *β* are intermediate variables. The plasma flow *F*_p_ and three intermediate parameters *M*, *α*, and *β* are first fitted from *C*_p_ and *C*_t_ using Equation 2. The *v*_p_, *K* ^trans^, and *v*_e_ are subsequently calculated using Equation 3.

### Pancreas segmentation and image analysis

A radiologist (LW), who has 11-year clinical experience in the reading of abdominal MRIs and was blinded to the histopathological diagnosis, evaluated all the MRI images. The margin of the pancreas for all subjects were drawn manually on the Multitasking DCE images. For PDAC images, the tumor boundary was identified by cross-referencing the non-contrast MRI protocols of the same imaging session and the clinical contrast-enhanced CT images acquired within 1 week before the study. The region of interest (ROI) of PDAC tumor was then defined on multiple slices within the boundary of tumor avoiding edges and vessels. The ROI of pancreas upstream and downstream were defined subsequently, if applicable. The ROI of the PDAC pancreas was a combination of the ROIs of PDAC tumor, upstream (if any), and downstream (if any). For CP and normal control pancreas, the ROI was maximized within the pancreas margin. As a summary, six types of tissues were defined: 1) PDAC tumor, 2) PDAC upstream, 3) PDAC downstream, 4) PDAC pancreas, 5) CP pancreas, and 6) normal control pancreas. The microcirculation parameters reported for each type of tissue for each case were the average of all voxels within the ROI.

### Statistical analysis

Statistical analysis was conducted in SPSS (Version 24, IBM, NY, USA). The descriptive statistics including mean and standard deviation (SD) were obtained for six type of tissues: PDAC pancreas, PDAC tumor, PDAC upstream, PDAC downstream, CP pancreas, and normal control pancreas. Analysis of variance (ANOVA) with Bonferroni correction was used to assess the multi-group comparison. The value of P< 0.05 was considered statistically significant. The performance of the microcirculation parameters in the differentiation of the tissues were assessed with receiver operating characteristic (ROC) analysis. The sensitivity, specificity, and area under the ROC curve (AUC) of each single microcirculation parameter and combination of all parameters were evaluated. An AUC of 0.5 to 0.6 suggests no discrimination, 0.6 to 0.7 is considered poor, 0.7 to 0.8 is fair, 0.8 to 0.9 is good, and > 0.9 is excellent (34).

### Comparison of quantitative DCE analysis versus conventional time-signal intensity curve analysis

For clinical multi-phase MRI, quantitative microcirculation parameters are unavailable due to the small number of DCE phases acquired and the low temporal resolution. Under this circumstance, time-signal intensity curve (TIC) approach serves as an alternative way to analyze the CA dynamics (22). It classifies the shape of the time-signal intensity curves into several categories based on the time to the peak and the wash-out patterns, as shown in **Supplementary Figure S1** in the supplementary materials. The enhancement patterns are useful to differentiate pathological tissues from normal. Zhang et al (22) reported that conventional multi-phase MRI with TIC analysis was able to differentiate mass-forming pancreatitis from PDAC. To compare the differentiation ability of the quantitative DCE analysis versus the TIC analysis, the high-temporal-resolution Multitasking DCE images were averaged to a temporal resolution of 18-second per phase and 6 key phases were chosen for analysis: pre-contrast, 18-second, 45-second, 75-second, 2.5-minute, 4-min post-contrast. The pattern of the signal intensity curves were classified into 5 types (18), as illustrated in **Supplementary Figure S1A** in the supplementary material: type I, a rapid rise to the peak at 18 s after injection; type II to V, a slower rise to a peak at 45s, 75s, 2.5 or 4 min after the injection, respectively. For each type of curve, two subtypes were defined based on the wash-out pattern (**Supplementary Figure S1B**): subtype-a, more than 10% signal decrease after reaching the peak; subtype-b, less than 10% signal decrease after the peak. Each tissue of each case was assigned to a category and the ROC analysis was performed to differentiate the tissues based on their categories. Subsequently, a significance test was performed to compare the AUC values produced by the ROC analysis using TIC versus using quantitative DCE approach according to DeLong test using MedCalc (MedCalc Software Ltd, Belgium).

## Results

### Demographics

**Figure 1:**
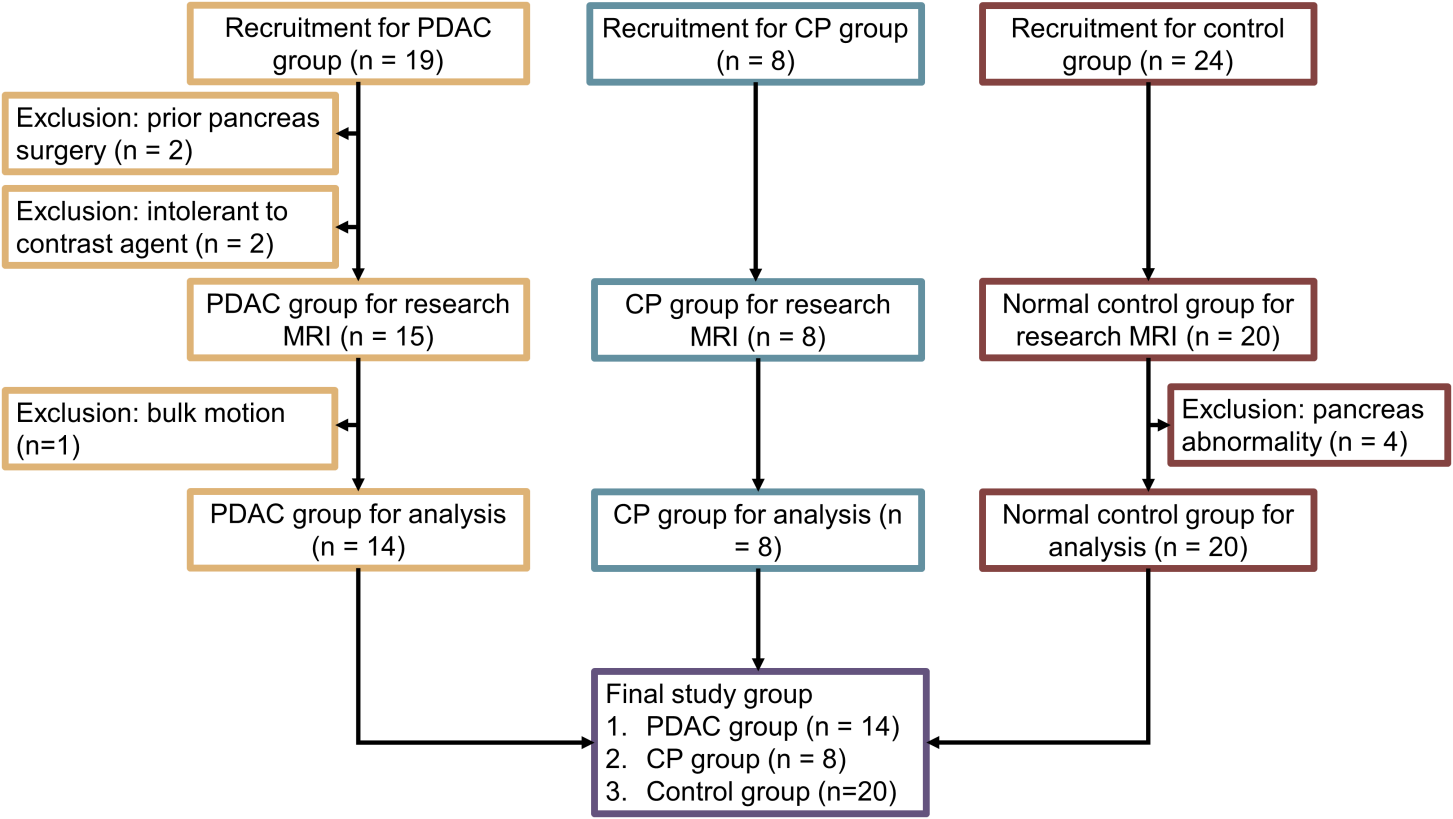
Flow chart for subject recruitment and grouping.

The demographics of this study are summarized in **Figure 1**. Among the 19 PDAC patients, two of them had undergone surgery on the pancreas in the past. Additionally, two others were not able to receive MRI contrast agent. Finally, another one patient had excess bulk motion during the study, yielding unreadable MR images. These patients were excluded, and the final group included 14 PDAC patients (51 to 77 years old, 7 females). The mean size of the tumors, defined as the largest diameter in axial CT images according to RECIST 1.1 criteria31, was 3.9 cm, ranging from 1.6 cm to 6.7 cm. Six tumors were in the pancreatic head, three in the pancreatic neck, three in the pancreatic body, and two in the pancreatic tail. The PDAC downstream was measurable in 10 cases, while the PDAC upstream was measurable in 9 cases. A total of 8 CP patients (30 to 72 years old, 4 females) underwent MRI imaging. Upon review of the images, all of them met the Cambridge criteria for CP. In addition, a total of 20 healthy subjects (23 to 60 years old, 9 females) were included as normal control group in the study.

### Quantifications of microcirculation parameters for different tissues

**Figure 2:**
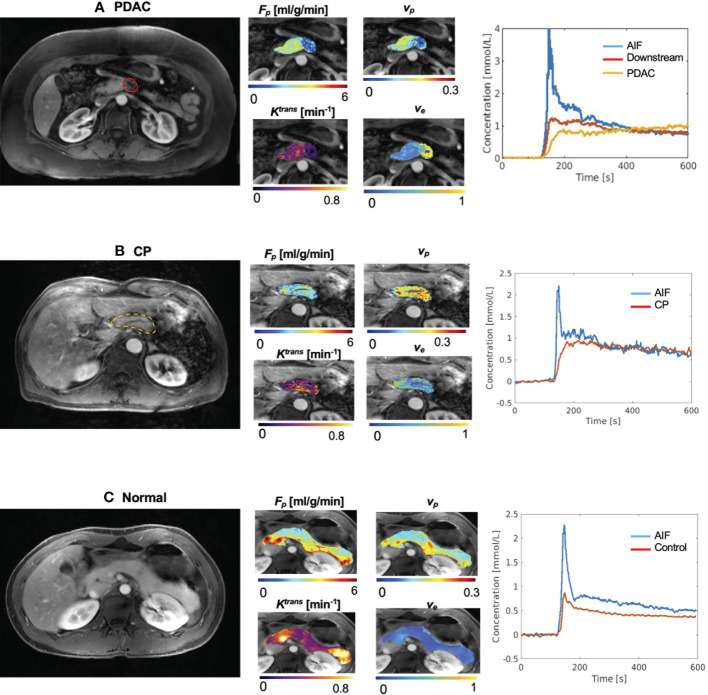
Example microcirculation parametric maps. **(A)** Example maps from 72-year-old patient with PDAC, whose tumor is located at the neck of pancreas. First panel shows a gray-scale Multitasking image at the arterial phase at the center slice of the tumor. The tumor was labeled by the red solid boundary. Downstream was visible in this slice. The second panel shows estimated microcirculation parametric maps. PDAC tumor showed lower F_p_, lower v_p_, and elevated v_e_ compared to downstream. The third panel shows the averaged contrast agent concentration curves for blood, PDAC tumor, and PDAC downstream. **(B)** Example maps from a 65-year-old patient with CP. The pancreas was labeled by the yellow dashed boundary. **(C)** Representative maps of a 32-year-old subject in the normal control group.

The microcirculation parameters were estimated successfully for all the involved subjects. **Figure 2** (A) shows the example microcirculation parametric maps from a 72-year-old PDAC patient with the tumor located at the neck of the pancreas, as labeled by red solid boundary on the gray-scale image. The CA concentration curve of PDAC tumor shows slower and progressive enhancement, while the concentration curve of downstream pancreas showed faster wash-in and moderate wash-out. Reduced F_p_, v_p_, K^trans^, and increased v_e_ was observed in PDAC tumor. **Figure 2B** is an example from a 65-year-old patient with CP, labeled by yellow dashed boundary. An example of normal control pancreas from a 32-year-old healthy subject is shown in **Figure 2C**.

**Figure 3:**
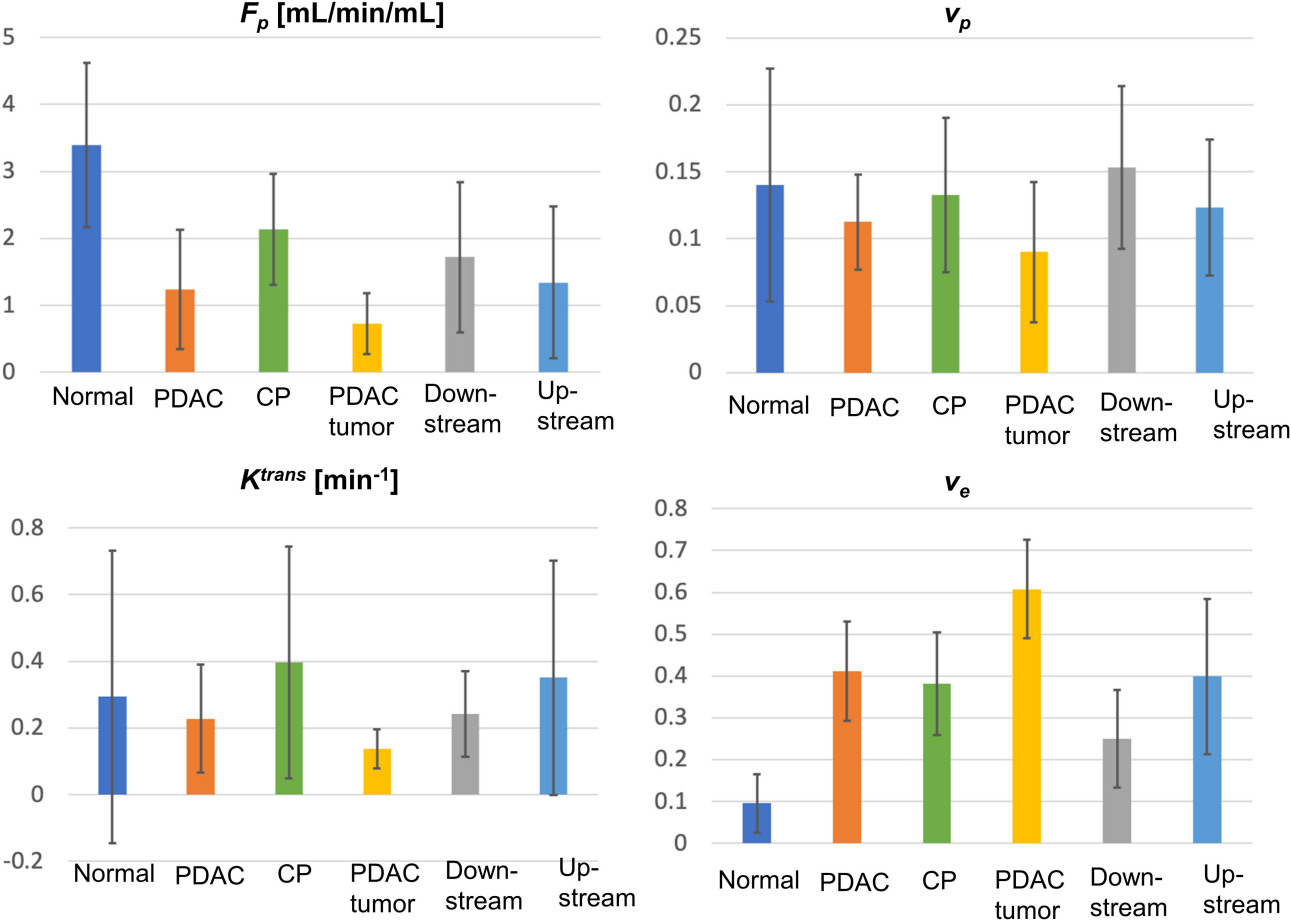
Bar graphs for the mean and standard deviation (error bar on top of each bar) for F_p_, v_p_, K^trans^, and v_e_ for all types of tissues (normal, normal control pancreas; PDAC, PDAC pancreas; CP, CP pancreas).

The mean and standard deviation measurement of F_p_, v_p_, K^trans^, and v_e_ for the six types of tissues are displayed in the bar graphs in **Figure 3**. The detailed mean values and standard deviations of each microcirculation parameter for each tissue are displayed in **Table 2**. The ANOVA test with Bonferroni correction of each microcirculation parameters between some pairs of tissues are listed in **Table 3**. The former half of **Table 3** shows the comparison of each pair of CP, PDAC pancreas, and normal control pancreas. With Bonferroni correction, significant differences were observed in F_p_ for CP versus PDAC pancreas (P = 0.015), and in F_p_ and v_e_ for CP versus normal control (P = 0.012,<0.001, respectively) and PDAC pancreas versus normal control (P<0.001,<0.001, respectively). The latter half of **Table 3** compares the measurements between CP versus PDAC tumor, downstream, and upstream. F_p_, K^trans^, and v_e_ showed significant differences between CP and PDAC tumor (P<0.001, = 0.012,<0.001, respectively); v_e_ was significantly different between CP and PDAC downstream (P =0.024); None of the microcirculation parameters showed significant differences between CP and PDAC upstream.

**Table 2:** The mean and standard deviation of F_p_, v_p_, K^trans^, and v_e_ for the six types of tissues.

	*F_p_* (mL/min/mL)	v_p_	K^trans^ (min^-1^)	v_e_
Control	3.39±1.23	0.14±0.09	0.29 ± 0.43	0.10±0.07
PDAC whole	1.24±0.89	0.11±0.04	0.23 ± 0.16	0.41±0.11
CP	2.13±0.83	0.13±0.06	0.40 ± 0.35	0.38±0.12
PDAC mass	0.72±0.04	0.09±0.05	0.14 ± 0.06	0.61±0.11
Downstream	1.71±1.21	0.15±0.06	0.24 ± 0.13	0.25±0.12
Upstream	1.34±1.13	0.12±0.05	0.35 ± 0.35	0.40±0.18

**Table 3:** The *P* value between some pairs of the tissues using one-way ANOVA analysis.

Comparison pairs		*F_p_* (mL/min/mL)	v_p_	K^trans^ (min^-1^)	v_e_
CP	PDAC pancreas	0.015*	0.327	0.136	0.583
CP	Normal control pancreas	0.012*	0.827	0.561	<0.001*
PDAC pancreas	Normal control pancreas	<0.001*	0.276	0.601	<0.001*
CP	PDAC tumor	<0.001*	0.093	0.012*	<0.001*
CP	PDAC downstream	0.383	0.454	0.175	0.024*
CP	PDAC upstream	0.125	0.732	0.795	0.830

* indicate statistical significance after Bonferroni correction.

### Prediction of type of tissue using microcirculation parameters

**Figure 4:**
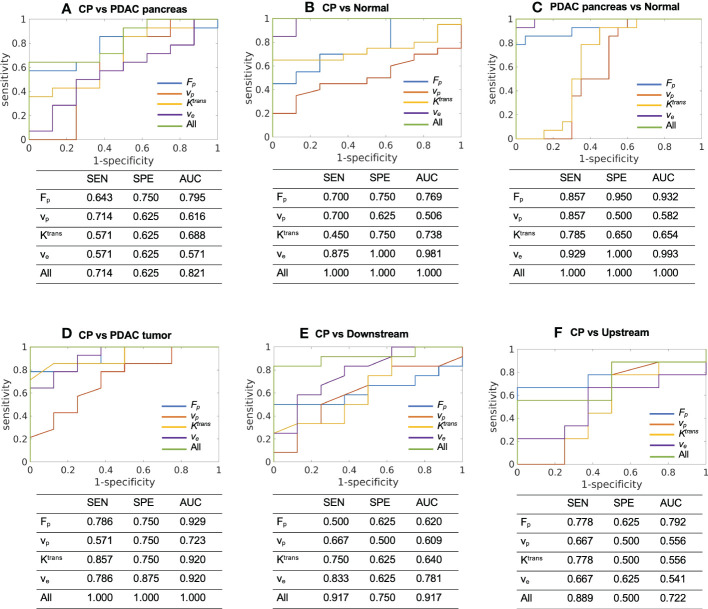
ROC curves and the sensitivity (SEN), specificity (SPE), and AUC to differentiate between **(A)** CP (N = 8) versus PDAC pancreas (N = 14), **(B)** CP (N = 8) versus normal control pancreas (N = 20), **(C)** PDAC pancreas (N = 14) versus normal control pancreas (N = 20), **(D)** CP (N = 8) versus PDAC tumor (N = 14), **(E)** CP (N = 8) versus PDAC downstream (N = 10), and **(F)** CP (N = 8) versus PDAC upstream (N = 9) using each single microcirculation parameter or the combination of all the four parameters. SEN, sensitivity; SPE, specificity; AUC, area under ROC curve.

The ROC analysis was successfully carried out to evaluate the performance of microcirculation parameters in differentiating CP versus other tissues using either each single parameter or a combination of the four parameters. In the differentiation of CP and PDAC pancreas (**Figure 4A**, F_p_ showed the highest accuracy (AUC [95% CI] = 0.795 [0.604 - 0.985]) as a single parameter; the combination of the four parameters produced improved differentiation ability with good AUC (0.821 [0.654 – 0.988]). In the differentiation of CP and normal control pancreas (**Figure 4B**), v_e_ showed the highest AUC (0.981 [0.938 – 1.000]) when using single parameter; the combination of the four parameters can differentiate all the cases of current study cohort with AUC = 1.000 [1.000 – 1.000]. In the differentiation of PDAC pancreas and normal control pancreas (**Figure 4C**), v_e_ showed the highest AUC (0.993 [0.974 – 1.000]) for single parameter; the combination of the four parameters can differentiate all the cases with AUC = 1.000 [1.000 – 1.000]. For CP versus PDAC tumor (**Figure 4D**), F_p_ (0.929 [0.824 – 1.000]), K^trans^ (0.920 [0.806 – 1.000]), and v_e_ (0.920 [0.805 – 1.000]) showed excellent differentiation ability when using a single parameter; the combination of all the four parameters can differentiate all the cases with AUC = 1.000 [1.000 – 1.000]. For CP versus PDAC downstream (**Figure 4E**), v_e_ showed the highest AUC for single parameter (0.781 [0.569 – 0.994]), while the combination of the four parameters showed increased accuracy with excellent AUC (0.917 [0.795 – 1.000]). For CP versus upstream (**Figure 4F**), F_p_ showed the fair AUC for single parameter (0.792 [0.558 – 1.000]), and the combination of the four parameters showed slightly reduced but still fair accuracy (0.722 [0.465 – 0.980]).

### Prediction of type of tissue using conventional TIC analysis

**Figure 5:**
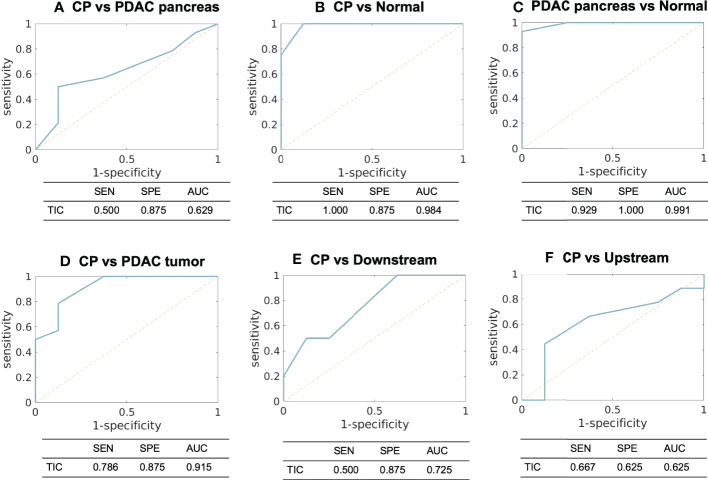
ROC curves and the sensitivity, specificity, and AUC using TIC analysis to differentiate between **(A)** CP versus PDAC pancreas, **(B)** CP versus normal control pancreas, **(C)** PDAC pancreas versus normal control pancreas, **(D)** CP versus PDAC tumor, **(E)** CP versus PDAC downstream, and **(F)** CP versus PDAC upstream. SEN, sensitivity; SPE, specificity; AUC, area under ROC curve; TIC, time-signal intensity curve.

For TIC analysis, the number of cases in each category for each type of tissue are summarized in **Table 4**. CP demonstrated the TIC of type II (n = 2), type III (n = 5), and type IV (n = 1); the PDAC pancreas demonstrated type II (n = 3), type III (n = 4), and type IV (n = 7), which were within the same range of CP; PDAC tumor demonstrated type III (n = 3), type IV (n = 4), and type V (n = 7), representing a slower enhancement. **Figure 5** displays the ROC plots to differentiate the CP versus PDAC pancreas (AUC [95% CI] = 0.629 [0.400 – 0.823], poor), CP versus normal control pancreas (0.984 [0.944 – 1.000], excellent), PDAC pancreas versus normal control pancreas (0.991 [0.968 – 1.000], excellent), CP versus PDAC tumor (0.915 [0.789 – 1.000], excellent), CP versus downstream (0.725 [0.468 – 0.905], fair), and CP versus upstream (0.625 [0.342 – 0.908], poor) with the sensitivity, specificity, and AUC listed under each plot.

**Table 4:** TIC category for each type of tissue.

Category	PDAC whole	CP	PDAC mass	Down-stream	Upstream	Control
I a	0	0	0	2	1	10
I b	0	0	0	0	0	5
II a	1	1	0	3	0	5
II b	2	1	0	0	1	0
III a	3	3	0	5	1	0
III b	1	2	3	0	2	0
IV a	4	0	3	0	4	0
IV b	3	1	1	0	0	0
V	0	0	7	0	0	0

TIC, time-signal intensity curve.

### Comparison of quantitative DCE analysis and conventional TIC analysis

**Table 5** lists the AUC from TIC, the highest AUC using a single microcirculation parameter from quantitative DCE, the AUC of combining all microcirculation parameters for the differentiation of each pair, and the P values using DeLong test to compare the performance of TIC analysis and quantitative DCE analysis. In most pairs, a single microcirculation parameter from quantitative DCE approach produced higher AUC than conventional TIC approach (except in the differentiation of CP versus normal control, where the AUC for TIC is 0.984 and the AUC from a single microcirculation parameter is 0.981). The combination of microcirculation parameters demonstrated higher AUC in all pairs when compared to the conventional TIC approach. The DeLong test indicated that quantitative DCE analysis performed significantly better in differentiating CP versus PDAC pancreas (P = 0.032) and CP versus PDAC downstream (P = 0.042).

**Table 5:** The comparison of the differentiation ability of TIC approach versus quantitative DCE approach.

Pairs		AUC of TIC	Highest AUC of a single microcirculation parameter	AUC of combined microcirculation parameters	P value
CP	PDAC pancreas	0.629	0.795	0.821	0.032*
CP	Normal control	0.984	0.981	1.000	0.353
PDAC pancreas	Normal control	0.991	0.993	1.000	0.353
CP	PDAC tumor	0.915	0.920	1.000	0.179
CP	Downstream	0.725	0.781	0.917	0.042*
CP	Upstream	0.625	0.792	0.722	0.380

For most pairs, the highest AUC produced by a single microcirculation parameter is higher than the AUC of TIC. The combination of the four microcirculation parameters outperforms the TIC analysis for all the pairs.

* indicates statistical significance.

## Discussions

The differential diagnosis between PDAC and CP remains an unmet clinical need. In terms of clinical factors, both diseases can have similar background histories such a history of alcohol and tobacco use, and similar clinical signs such as weight loss, chronic abdominal pain, anorexia, and diabetes (8). For blood test, the best-established biomarker for PDAC diagnosis is carbohydrate antigen 19-9 (CA19-9), a Lewis antigen of the MUC1 protein class. Unfortunately, CA19-9 can also be elevated in patients with CP, yielding a distinction no better than 65% (35). Imaging is the most common approach to diagnose these diseases. Contrast-enhanced CT and multi-phasic contrast-enhanced MRI have shown high sensitivity and high specificity for the diagnosis of CP or PDAC solely (36, 37). However, the shared imaging findings make the differential diagnosis a complicated issue. The common imaging features include generalized parenchymal glandular atrophy, diffuse pancreatic calcifications, dilation of the main pancreatic duct, hypo-attenuation on contrast-enhanced CT (38), and hypo-enhancement on multi-phasic contrast-enhanced MRI. EUS and EUS-guided fine needle aspiration (FNA) have high sensitivity and specificity in the detection of PDAC and CP (39). However, studies have shown that the sensitivity drops significantly to only 50–75% in patients with chronic pancreatitis (40, 41) due to shared pathological features (17).

In recent years, advanced techniques have been developed to improve the diagnosis and differentiation of PDAC and CP. New blood biomarkers including plasma suPAR (42) and a bunch of metabolic markers (43) have shown promises in the differentiation of the two diseases. Perfusion CT has been used for the diagnosis and differentiation of PDAC (44) and CP (45, 46) with positive results, but remains in the research phase for pancreas due to higher radiation dose and limited field of view. In MRI, non-contrast techniques including diffusion-weighted imaging and T1 mapping, and contrast-enhanced techniques with more dynamic phases and TIC analysis (22) also show promising differential ability of the two diseases (12, 45). These new techniques are non-invasive approaches with clinical promises, but still need to be validated on larger cohorts of patients.

In this work, we investigated the differential ability of tissue microcirculation parameters estimated from Multitasking DCE technique. The microcirculation properties carry crucial information about disease characteristics, progression, and regression. The alteration of microcirculation properties usually precedes morphological changes (47–49), providing a pathway for early detection, staging, and treatment monitoring. DCE MRI has the potential to capture the microcirculation properties by tracking the contrast agent kinetics within the tissues, but has been limited by the demanding sampling requirements. The pathological lesions usually bear high-level heterogeneity within the structure, which requires adequate coverage and high spatial resolution to capture the spatial variation. On the other hand, high temporal resolution is required to accurately track the kinetics of contrast agent within the tissues. Previous studies have demonstrated that a temporal resolution of least 10 seconds is necessary to depict tumor enhancement dynamics (50), and 1-3 seconds to capture the dynamics of AIF (51, 52). Furthermore, respiratory motion remains a major challenge and can further degrade the image quality for pancreas imaging.

The recently-proposed Multitasking DCE technique is a promising solution to resolve the abovementioned limitations (28–31, 53–55). The technique is capable of resolving respiratory motion, achieving a free-breathing acquisition for 10-minutes to capture the contrast agent kinetics. It enables entire-abdomen coverage, clinical-sufficient spatial resolution, and 1-second temporal resolution simultaneously, allowing for the capture of spatial variation and temporal kinetics. Consequently, quantitative DCE analysis can be performed to estimate the microcirculation parameters. In this work, the two-compartment exchange model was used, and four independent microcirculation parameters were estimated: F_p_, representing tissue blood flow, v_p_, correlated with the microvascular density, K^trans^, which has a joint effect of blood flow and permeability-surface area product, and v_e_, which is correlated with fibrosis content. Compared with normal control pancreas, the PDAC tumor, PDAC pancreas and CP all showed significantly lower F_p_ (P<0.001,<0.001, 0.012, respectively) and higher v_e_ (P<0.001,<0.001,<0.001, respectively), consistent with their pathological characteristics including reduced blood flow and high fibrosis replacement.

These microcirculation parameters showed strong ability in the differentiation of CP versus PDAC tumor. F_p_, K^trans^, and v_e_ demonstrated significant difference between CP and PDAC tumor (P<0.001, 0.012,<0.001, respectively); the AUC using these three parameters individually to differentiate CP versus PDAC tumor are all above 0.9 (0.929, 0.920, 0.920, respectively), representing excellent differential ability. The non-tumoral part of PDAC is frequently associated with secondary inflammatory changes (11, 56). This associated pancreatitis happens more frequently in the upstream of PDAC due to the obstruction of pancreatic duct (57, 58). The differential ability of the microcirculation parameter between CP and non-tumoral tissues in PDAC were also evaluated. For CP versus PDAC downstream, v_e_ showed significant difference (P = 0.024) and produced the highest AUC (0.781) with a single parameter; the combination of all the four parameters demonstrated excellent differential ability with an AUC of 0.971. For CP versus PDAC upstream, F_p_ showed a visible reduction in the upstream as displayed in **Figure 3**, but none of the microcirculation parameters had significant difference between the two tissues. A major reason could be the large standard deviation from the small sample size and varied diseases grade or severity. The ROC analysis showed a fair AUC of 0.792 using F_p_ and 0.722 when combining of all the four parameters. These results indicate that the microcirculation parameters, especially F_p_, has a great potential to differentiate CP with PDAC upstream.

Furthermore, the evaluation of differential ability between CP and whole PDAC pancreas were performed. F_p_ was significantly different between CP and PDAC pancreas (P = 0.015) and showed a fair and close to good AUC of 0.795. Combing of all parameters presented a good differentiation with an AUC of 0.821. The ability to differentiate CP versus whole PDAC pancreas has great utility in clinical context. It provides the possibility to identify patients with PDAC without the accurate localization of tumor.

To demonstrate the advantages of the quantitative Multitasking DCE technique, the comparison with the conventional TIC approach was also performed. The TIC approach showed excellent accuracy to differentiate CP versus PDAC tumor (AUC = 0.915), CP versus normal control pancreas (AUC = 0.984), and PDAC pancreas versus normal control pancreas (AUC = 0.991). These results are comparable to the highest AUCs produced by a single microcirculation parameter but lower than the AUCs from the combination of all microcirculation parameters. For CP versus downstream, TIC produced fair accuracy with AUC of 0.725, while the microcirculation parameters showed excellent differential ability with AUC of 0.917. For CP versus upstream, TIC produced poor differentiation ability with AUC of 0.625, perhaps due to the similar enhancement pattern of CP and PDAC upstream with associated pancreatitis. The quantitative DCE approach, on the other hand, can capture more dynamic information and improve the differentiation ability to an AUC of 0.792. For CP versus PDAC pancreas, TIC performed poorly with AUC of 0.629. while quantitative DCE approach presented a good differentiation (AUC = 0.821) when using all microcirculation parameters. The comparison demonstrated that Multitasking DCE with quantitative DCE analysis outperformed the conventional TIC approach and can potentially improve the differentiation between CP versus PDAC.

Another intriguing potential of the quantitative Multitasking DCE technique is to evaluate and predict the treatment outcome of PDAC and CP (24, 59, 60). Most of the therapies affect tumor microvasculature and thus the microcirculation properties, altering tumor blood flow, microvascular density, and extravascular extracellular distribution. By identifying the changes in microcirculation properties with quantitative Multitasking DCE, there is a great potential to predict the treatment effect at early stage and individualize the therapy regimen.

Our study has several limitations. First, the sample sizes for all groups were relatively small, which may affect the statistical outcome. The ability to differentiate CP versus PDAC of Multitasking DCE need to be validated on larger patient cohort. Second, the PDAC and CP groups included a variety of tumor stages or disease severity. Sub-group analysis based on tumor grade or disease severity was not possible due to the small sample size. This variation can be a major factor contributing to the wide standard deviation of the microcirculation parameters. Third, all the PDAC patients had undergone neoadjuvant chemotherapy at the time of the study, which may change the tissues properties. Future studies on treatment-naïve PDAC patients will be performed. Finally, the resection specimens were not available in this pilot study. The correlation between the microcirculation parameters and the histological markers including the microvascular density and fibrosis were not accessible in this work. With the promising preliminary results, future studies with the correlation between pathological details and imaging parameters will be performed on untreated PDAC and CP patients.

## Conclusion

A novel Multitasking DCE MRI technique with quantitative analysis of microcirculation parameters was performed to differentiate PDAC and CP. The combination of the microcirculation parameters showed strong ability to different CP from normal control pancreas, PDAC pancreas, PDAC tumor, PDAC downstream, and PDAC upstream, and superior performance compared to conventional TIC analysis approach. Multitasking DCE appears to be a promising clinical tool for the differentiation of CP from PDAC on a quantitative and objective basis.

## Data availability statement

The data will be available upon request with de-identification; the analytic methods are available upon request to the corresponding author.

## Ethics statement

The studies involving human participants were reviewed and approved by Cedars-Sinai Medical Center. The patients/participants provided their written informed consent to participate in this study.

## Author contributions

NW contributed to the data acquisition, analysis and interpretation, and article writing. SG, SP, SL, and AH contributed to the patient recruitment and study design. YX, AC, CW, SM, ZF, LW contributed to the data analysis and interpretation. DL contributed to the conception and study design. All authors contributed to the article and approved the submitted version.
