# Supplementary material for: Multitasking dynamic contrast enhanced magnetic resonance imaging can accurately differentiate chronic pancreatitis from pancreatic ductal adenocarcinoma
Source: Front Oncol. 2023 Jan 6;12:1007134. doi: 10.3389/fonc.2022.1007134 (PMC9853434; doi:10.3389/fonc.2022.1007134)
Supplement: Supplementary file 1 [file DataSheet_1.docx]

Supplementary Material

# Supplementary Figure


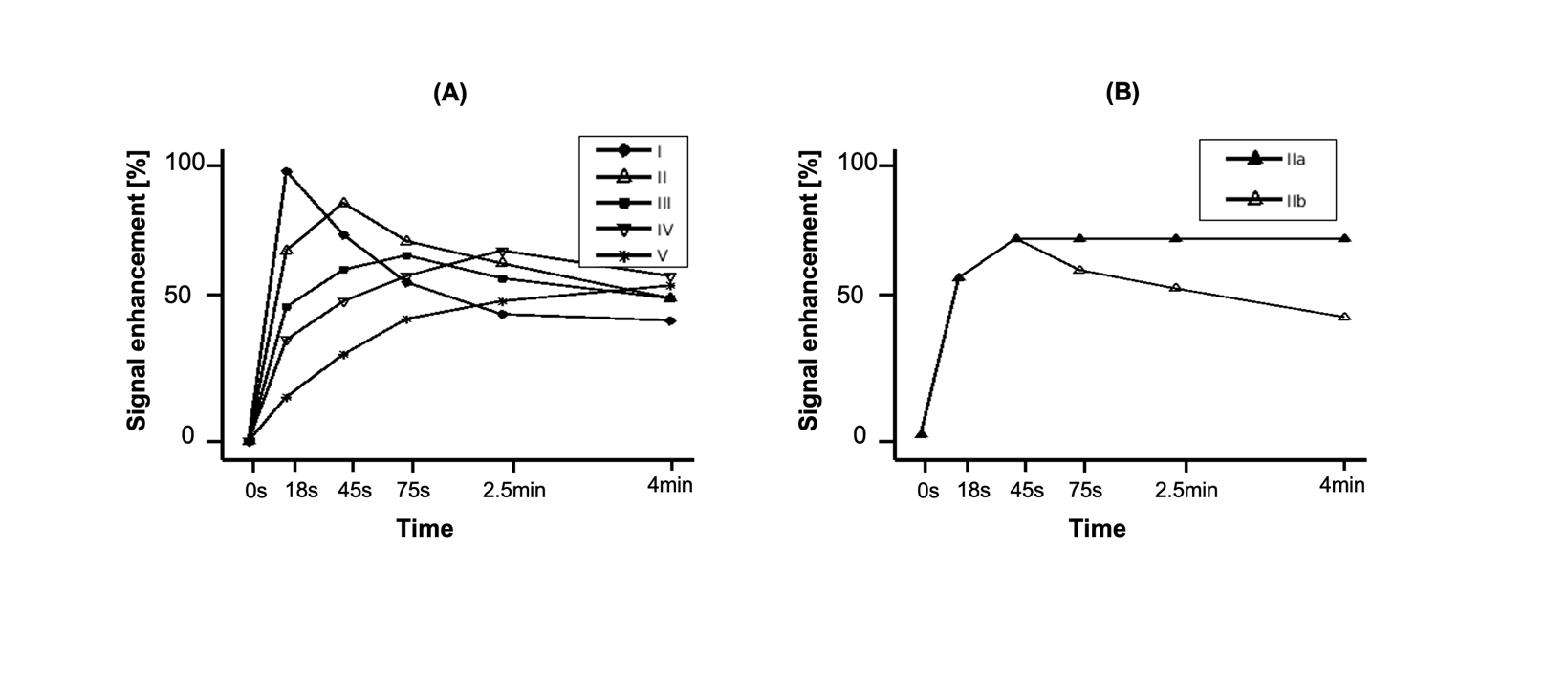


Supplementary Figure S1: TIC types. (A) type I, a rapid rise to a peak at 18 s after injection; type II to V, with a slower rise to a peak at 45s, 75s, 2.5 or 4 min after the injection. (B) subtype-a, wash-out; subtype-b, plateau.
